# HSQC-TOCSY Fingerprinting-Directed Discovery of Antiplasmodial Polyketides from the Marine Ascidian-Derived *Streptomyces* sp. (USC-16018)

**DOI:** 10.3390/md16060189

**Published:** 2018-05-30

**Authors:** Larissa Buedenbender, Luke P. Robertson, Leonardo Lucantoni, Vicky M. Avery, D. İpek Kurtböke, Anthony R. Carroll

**Affiliations:** 1 Environmental Futures Research Institute, School of Environment and Science, Griffith University, Gold Coast Campus, QLD 4222, Australia; larissa.buedenbender@griffithuni.edu.au (L.B.); luke.robertson2@griffithuni.edu.au (L.P.R.); 2 Griffith Institute for Drug Discovery, Griffith University, Brisbane, QLD 4111, Australia; l.lucantoni@griffith.edu.au (L.L.); v.avery@griffith.edu.au (V.M.A.); 3 GeneCology Research Centre, Faculty of Science, Health, Education and Engineering, University of the Sunshine Coast, Maroochydore, QLD 4558, Australia

**Keywords:** actinomycetes, *Streptomyces*, ascidian-associated actinomycetes, *Symplegma rubra*, polyketide, ansamycin derivative, herbimycin, antiplasmodial activity

## Abstract

Chemical investigations on the fermentation extract obtained from an ascidian-derived *Streptomyces* sp. (USC-16018) yielded a new ansamycin polyketide, herbimycin G (**1**), as well as a known macrocyclic polyketide, elaiophylin (**2**), and four known diketopiperazines (**3**–**6**). The structures of the compounds were elucidated based on 1D/2D NMR and MS data. The absolute configuration of **1** was established by comparison of experimental and predicted electronic circular dichroism (ECD) data. Antiplasmodial activities were tested for the natural products against chloroquine sensitive (3D7) and chloroquine resistant (Dd2) *Plasmodium falciparum* strains; the two polyketides (**1**–**2**) demonstrated an inhibition of >75% against both parasite strains and while **2** was highly cytotoxic, herbimycin G (**1**) showed no cytotoxicity and good predicted water solubility.

## 1. Introduction

Malaria is a vector-borne disease caused by protozoan parasites of the genus *Plasmodium*, which results in more than 400,000 deaths annually [1]. The life cycle of the malaria parasite involves two hosts. The disease is contracted when an *Anopheles* mosquito feeds on human blood and simultaneously injects sporozoites into the bloodstream. The sporozoites are then carried to the liver, where they reproduce asexually to give rise to thousands of merozoites that spread through the body via the bloodstream [2,3]. During this intraerythrocytic cycle, the clinical manifestations of the disease appear, and the human host experiences strong fevers and chills [3,4]. Within the red blood cells, merozoites develop into ring forms and then trophozoites, which mature to form multinucleated schizonts that eventually burst and generate new merozoites. A small proportion of trophozoites produce male and female gametocytes. The mature gametocytes are transmitted back to a mosquito as it takes a blood meal. Within the gut of the mosquito, they undergo gametogenesis and fertilization, then develop into oocysts, which asexually produce new sporozoites. These migrate to the mosquito’s salivary glands and the life cycle of the parasite begins again once the mosquito feeds on a new human host [3]. The drug resistance against the currently available antimalarials is a major concern [5] and, therefore, new drugs with novel targets are urgently needed.

Macrolide polyketides have been considered as possible candidates for antimalarials [6]. For instance, the macrolide compound azithromycin exhibits antiplasmodial activity by targeting the relict plastid in the *Plasmodium* parasite and inhibiting protein synthesis in this organelle [7]. The ansamycin polyketide, geldanamycin, inhibits heat shock proteins, which are highly expressed in the early blood stage of the malaria parasite and thus inhibits the growth of the parasite [8]. Actinomycetes are a class of bacteria known to produce diverse macrolide polyketides. In order to further characterise new antiplasmodial natural products belonging to this chemical class, we targeted microbial isolates from underexplored ecological niches and 120 actinomycetes were isolated from Australian ascidian species [9].

**Figure 1:**
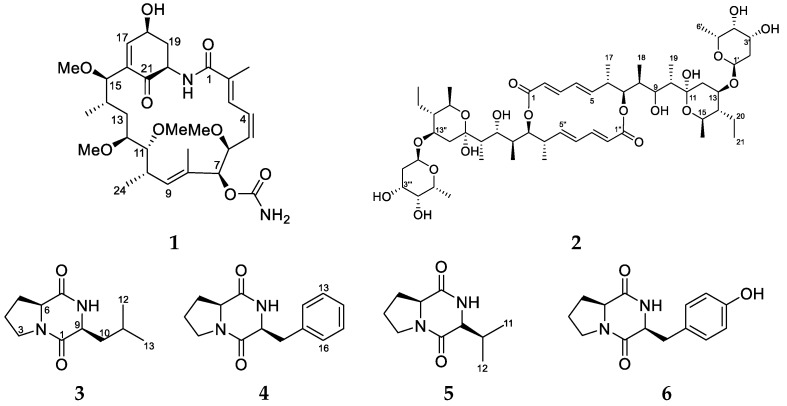
The polyketides and peptides isolated from the ascidian-associated *Streptomyces* sp. (USC-16018): herbimycin G (**1**); elaiophylin (**2**); l-Pro-l-Leu (**3**); l-Pro-l-Phe (**4**); l-Pro-l-Val (**5**); l-Pro-l-Tyr (**6**).

A new approach utilising two-dimensional heteronuclear single quantum coherence—total correlation spectroscopy nuclear magnetic resonance (HSQC-TOCSY NMR) profiles and biological activity data [10] allowed us to target antiplasmodial polyketide-producing strains based on identified regions of interest in the NMR spectrum that corresponded to correlations between upfield methyl proton resonances (*δ*_H_ 0.50–2.00) and carbon resonances between *δ*_C_ 60–80 ppm, which were associated with polyketide-type compounds. Based on this approach, *Streptomyces* sp. (USC-16018) derived from the colonial ascidian *Symplegma rubra* was selected because its crude extract HSQC-TOCSY NMR profile indicated the presence of several polyketide resonances (Supplementary Material, Figure S1). Furthermore, the extracts exhibited a 97% inhibition of *Plasmodium falciparum* at 0.2 μg/mL. The *Streptomyces* sp. was fermented on a larger scale and the purification of the ethyl acetate (EtOAc) extract derived from this culture resulted in the isolation of two antiplasmodial polyketides, herbimycin G (**1**), a new geldanamycin analogue, and the known antibiotic elaiophylin (**2**), as well as four known diketopiperazines (**3**–**6**) (Figure 1).

## 2. Results and Discussion

Fermentations of *Streptomyces* sp. (USC-16018) were scaled up to 55 culture plates for two solid media, oatmeal agar (OMA) and glucose-starch-tryptone media (GST), which yielded 1.6 g and 0.8 g of extract, respectively, after the extraction with EtOAc. The extracts were purified by repeated reversed-phase C_18_ high-performance liquid chromatography (HPLC) and the analysis of fractions by ^1^H NMR led to the identification of two polyketides (**1** and **2**) and four diketopiperazine compounds (**3**–**6**).

Herbimycin G (**1**) was isolated as a dark brown solid with a UV maximum at 245 nm. A sodiated molecule ion at *m*/*z* 601.3111 [M + Na]^+^ obtained from high resolution-electrospray ionisation-quadrupole time of flight-mass spectrometry (HR-ESI-QTOF-MS) together with ^1^H and ^13^C NMR data suggested that **1** had a molecular formula of C_30_H_46_N_2_O_9_. The ^1^H NMR spectrum of **1** in DMSO-*d*_6_ had resonances that could be assigned to an amide proton at *δ*_H_ 8.02 (d, *J* = 9.4 Hz); five olefinic proton resonances at *δ*_H_ 7.13 (dd, *J* = 12.2 Hz, 1.2 Hz), *δ*_H_ 6.65 (ddd, *J* = 3.3 Hz, 1.9 Hz, 1.4 Hz), *δ*_H_ 6.48 (ddd, *J* = 12.2 Hz, 10.9 Hz, 1.4 Hz), *δ*_H_ 5.65 (dd, *J* = 10.9 Hz, 7.9 Hz), and *δ*_H_ 5.20 (d, *J* = 7.7 Hz); and an oxygenated methine at *δ*_H_ 4.68 (dddd, *J* = 10.6 Hz, 5.1 Hz, 4.7 Hz, 2.5 Hz) that correlated to a hydroxy resonance at *δ*_H_ 5.56 (d, *J* = 5.1 Hz) in the correlation spectroscopy (COSY) spectrum. A further five oxygenated methines were observed at *δ*_H_ 4.56 (dd, *J* = 7.9 Hz, 1.4 Hz), *δ*_H_ 5.16 (s), *δ*_H_ 3.15 (m), *δ*_H_ 3.33 (m), and *δ*_H_ 4.15 (d, *J* = 1.4 Hz), and four methoxy-resonances occurred between *δ*_H_ 3.13–3.35 ppm. The ^1^H NMR spectrum contained an additional three methines at *δ*_H_ 4.55, *δ*_H_ 2.45, *δ*_H_ 1.51; two methylene pairs *δ*_H_ 1.55 and *δ*_H_ 1.41, as well as *δ*_H_ 1.96 and *δ*_H_ 2.26, and four methyl signals *δ*_H_ 1.84 (d, *J* = 1.2 Hz), *δ*_H_ 1.62 (s), *δ*_H_ 0.94 (d, *J* = 6.7 Hz), and *δ*_H_ 0.69 (d, *J* = 6.7 Hz). The ^13^C and heteronuclear single quantum correlation (HSQC) NMR data showed 30 carbon resonances (Table 1). This data suggested that **1** was a close analogue to previously reported herbimycins, containing an identical macrolactam ring to herbimycins A, D, E, and 17,19-dimethylthioherbimycin A (Supplementary Material, Figure S2) [11,12,13]. The structural differences arose through different substitution of the benzoquinone moiety between C-15 and the amide proton. In herbimycin G (**1**), the loss of an olefinic methine was observed while a methylene (*δ*_H_ 1.96, *δ*_H_ 2.26/*δ*_C_ 39.2) and methine (*δ*_H_ 4.55/*δ*_C_ 53.2) were gained. The COSY and HSQC analyses revealed an isolated spin system—1-hydroxy-3-amino-propyl—which included the exchangeable amide proton (*δ*_H_ 8.02). Total correlation spectroscopy (TOCSY) correlations from the olefinic proton resonating at *δ*_H_ 6.65/*δ*_C_ 149.9 to all protons of this spin system implied that the olefinic proton was part of the system. Analysis of the heteronuclear multiple-bond correlation (HMBC) spectrum confirmed that the olefinic carbon (C-17) was *alpha* to the hydroxyl. The deshielded carbon resonance of C-17 indicated another electron withdrawing group *beta* to the double bond proton and a strong HMBC correlation from H-17 to a carbon at *δ*_C_ 197.1 determined it to be a carbonyl group. H-15 (*δ*_H_ 4.15) showed an additional two unassigned HMBC correlations to the olefinic carbon C-17 as well as a quaternary carbon at *δ*_H_ 134.0, linking the spin systems to the macrolactam ring at C-15. As one of the methylene protons (H-19b) also showed a correlation to the carbonyl resonance, it was concluded that this fragment consisted of a 4-hydroxyl-cyclohexenone system. The final structure was consistent with the sodium adduct ion *m*/*z* 601.3111 [M + Na]^+^ as well as the degrees of hydrogen deficiency calculated from the molecular formula.

Previously reported geldanamycins and herbimycins contained a 2,6-disubstituted-*p*-benzoquinone ring or a hydroquinone ring bonded to the macrolactam system, whereas herbimycin G (**1**), contains a 2,6-disubstituted 4-hydroxyl-cyclohexenone ring and, therefore, has an additional two stereogenic centres. Consequently, a ROESY spectrum was obtained in order to elucidate the relative configuration of the nine stereogenic centres in **1**. ROESY correlations between H-6 (*δ*_H_ 4.57) and H-7 (*δ*_H_ 5.16) showed that the two protons were *cis* to each other, indicating that the *O*-methyl at C-6 and the carbamate group attached to C-7 were on the *β*-face of the molecule. Based on the ROESY exchange (antiphase to the ROESY correlations) correlations, the exchangeable carbamate NH_2_ protons could be assigned as *δ*_H_ 6.30 and *δ*_H_ 6.66. A weak long-range correlation between H-7 and the methyl protons H-24 at *δ*_H_ 0.94 established the relative configuration of the two-spin system relative to C-7. The methyl protons at *δ*_H_ 0.94 also correlated to the methoxy protons at *δ*_H_ 3.35, and H-13a (*δ*_H_ 1.41), H-14 (*δ*_H_ 1.51), and H-15 (*δ*_H_ 4.15) were assigned to the *α*-face of the molecule. A weak ROESY correlation from H-15 to H-18 (*δ*_H_ 4.68) allowed for the determination of the configuration of the 4-hydroxyl-cyclohexenone system relative to the macrolactam ring.

**Figure 2:**
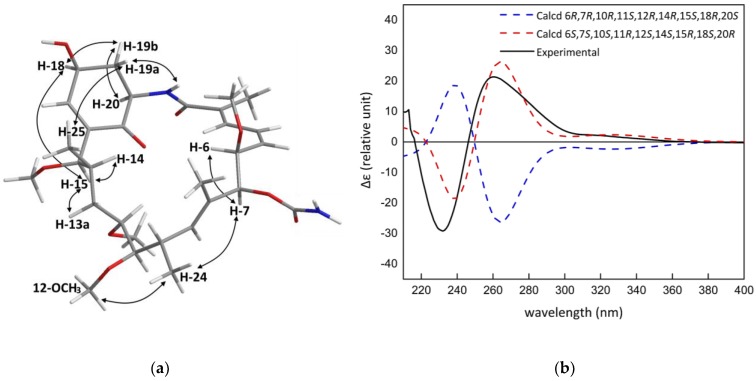
(**a**) The key ROESY correlations of **1**; (**b**) the comparison of the experimental electronic circular dichroism (ECD) spectrum of **1** with those calculated at the B3LYP/6-31G(d) level.

Further strong correlations between H-18 and H-19b (*δ*_H_ 2.26) to H-20 (*δ*_H_ 4.55), indicated their position to be on the *α*-face. The hydroxyl group, H-19a (*δ*_H_ 1.96), and the amide proton (*δ*_H_ 8.02) faced in the *β*-direction. No COSY correlation was observed between H-17 and H-18, indicating that the two protons were at an angle close to 90° and the small long-range couplings (*J* = 1.8 Hz) between H-17 and H-19b suggested a “*W*” arrangement. The ROESY correlations between the methyl resonating at *δ*_H_ 0.69 and *δ*_H_ 1.96 indicated that the macrolactam folded over the 4-hydroxyl-cyclohexenone. The two-dimensional structure was therefore established and redrawn in Maestro 2016 (Maestro Technologies, Inc., Trenton, NJ, USA) to perform a Monte-Carlo Multiple Minimisation (MCMM) search using the OPLS2005 force field (Schrödinger, New York, NY, USA) to generate the energy minimised conformation of **1** (Figure 2a). The previously observed ROESY correlations were compared to the energy-minimised 3D structure of **1**, confirming its relative configuration. The absolute configuration of **1** was determined by comparison of its experimental and predicted electronic circular dichroism (ECD) spectra, calculated using time-dependent density functional theory (TDDFT). A conformational search using MacroModel (Version 10.8, Schrödinger, LLC, New York, NY, USA) was performed on the energy minimised structure of **1** using the force field parameters as above with extended torsional sampling options. All structures within a 10.0 kJ/mol energy window were saved, generating 13 conformers. Geometry optimisation and Gibbs free energy calculations using Gaussian 16 (Revision A.03, Gaussian, Inc., Wallingford, CT, USA) at the B3LYP/31G(d) energy level was then performed on each of the conformers. Following this, the ECD spectra of each of the geometry optimised conformers was calculated using Gaussian 16 at the same theoretical level. A Boltzmann-weighted ECD spectrum was then calculated using SpecDis (Version 1.7, Würzburg, Germany) The experimental ECD spectrum of **1** showed good agreement with the calculated ECD of (6*S*,7*S*,10*S*,11*R*,12*S*,14*S*,15*R*,18*S*,20*R*)-**1** (Figure 2b).

The configuration of the macrolactam ring is consistent with herbimycin analogues previously reported in the literature, but herbimycin G (**1**) is the first example of an ansamycin compound that contains a 2,6-disubstituted 4-hydroxyl-cyclohexenone ring. A total of eleven herbimycin analogues, including herbimycin G, have now been reported from the *Streptomyces* sp. strains (Supplementary Material, Figure S2) [11,12,13,14,15,16]. Benzoquinone ansamycins have been identified as potent inhibitors of the Hsp90, an important cancer target and, more recently, a novel target for antimalaria drugs. Geldanamycin had reached clinical trials as an anticancer drug, however, the compound had low water solubility and exhibited high liver toxicity. As a result, a number of synthetically modified geldanamycin analogues were made and among those tanespimycin (17-N-allylamino-17-demethoxygeldanamycin, 17-AAG) advanced to phase three in anticancer clinical trials for multiple myeloma [17]. *In vitro* studies have found that geldanamycin equally inhibits the growth of chloroquine-sensitive and chloroquine-resistant *Plasmodium falciparum* strains (IC_50_ 20 nM) [18]. The 17-AAG analogue was reported to be effective against *Plasmodium yoelii* in mouse models when injected intra-peritoneal in doses of 7.2 mg/kg. However, while a single dose of chloroquine was sufficient to fully clear the animals from the parasites, a second dose of 17-AAG was needed [8]. In the same study, 17-AAG-treated mice that were reinfected with *P. yoelii* showed low percentages of parasitemia, which completely cleared after 9 days; the chloroquine-treated mice took a week longer to recover from the parasites [8]. Such reports suggest that ansamycin analogues may be good candidates for antiplasmodial drugs. Due to the low water solubility and high liver toxicity of geldanamycin, it is of great interest to find new water soluble and non-cytotoxic drug candidates to treat malaria infections.

**Figure 3:**
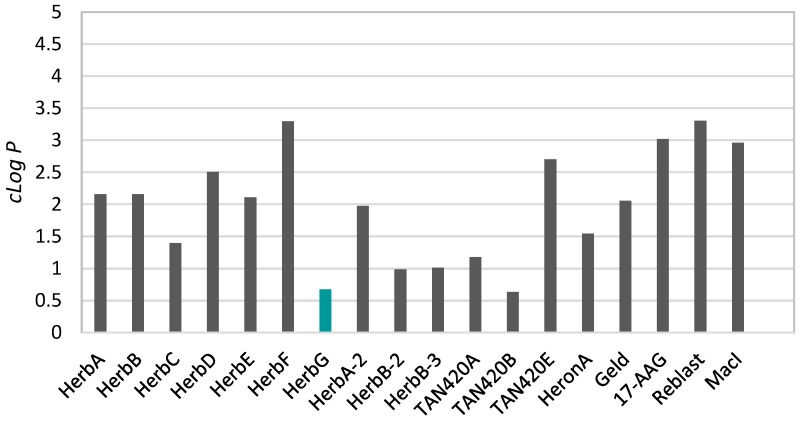
The analysis of the cLog*P* values of herbimycins and related ansamycin compounds (note: structures and keys for abbreviations for the presented compounds are given in the Supplementary Material Table S1 and Figure S2).

In herbimycin G (**1**), the benzoquinone was reduced to a 4-hydroxyl-cyclohexenone and, thus, potentially has better water solubility. Therefore, we assessed the cLog*P* and other Lipinski parameters of all known herbimycins, as well as several related ansamycin compounds (Figure 3; Supplementary Material, Table S1). Compared to geldanamycin, most herbimycin analogues, except for herbimycin D, F, and TAN-420E, exhibited similar or lower cLog*P* values. Herbimycin G (**1**) and TAN-420B had the lowest cLog*P*—0.68 and 0.63, respectively—indicating better water solubility. The molecular weights of all assessed herbimycin and geldanamycin compounds were non-compliant with the Lipinski rule of five that uses four physiochemical properties (molecular weight < 500 Da, partition coefficient cLog*P* < 5, hydrogen bond donors < 5, and hydrogen bond acceptors < 10) to assess drug-likeness [19]. Macrocycles frequently violate the rule of five but, nonetheless, have been successful as clinical leads (such as rifamycin) or candidates (such as erythromycin) and have made their way to the market [20]. This is due to their high binding affinity, stability, and specificity for a molecular target [20].

In addition to the new herbimycin analogue, EtOAc extracts from the *Streptomyces* sp. (USC-16018) grown on the GST media yielded the known polyketide, elaiophylin (**2**), and four diketopiperazines (**3**–**6**). The obtained spectroscopic NMR data for **3**–**6**, were compared to the literature and Cyclo-l-Pro-l-Leu (**3**), Cyclo-l-Pro-l-Phe (**4**), Cyclo-l-Pro-l-Val (**5**), and Cyclo-l-Pro-l-Tyr (**6**) were identified [21,22]. Additionally, a positive low resolution–electrospray ionisation–mass spectrometry (LR-ESI-MS) protonated molecular ion at *m*/*z* 1024.65 was observed and the molecular formula for **2** was established to be C_54_H_88_O_18_. Based on the ^1^H, COSY, HSQC, and HMBC spectra, a partial structure was elucidated containing 44 protons, 27 carbons, and 10 oxygen atoms. This only accounted for half the number of carbons and hydrogens, suggesting that **2** was a symmetric dimer. The comparison of these NMR data with the carbon data documented in the literature identified **2** as the known antibiotic compound elaiophylin [23]. Several *Streptomyces* species are known to produce elaiophylin and geldanamycin compounds. Recently, the complete genome mining of four geldanamycin producing *Streptomyces* sp. strains identified up to 56 putative biosynthetic gene clusters and found highly similar geldanamycin and elaiophylin gene clusters in all the strains [24]. Geldanamycin and herbimycin have the same ansamycin carbon skeleton. It is therefore assumed that both biosynthetic pathways exhibit very similar polyketide synthase (PKS) activity. In fact, Rasher and co-workers [25] proposed that both compounds are “assembled from the 3-amino-5-hydroxybenzoic acid (AHBA) starter unit by successive condensation of two-carbon building blocks in seven chain-elongation steps that use one malonyl, four methylmalonyl, and two methoxymalonyl extender units” and structural differences are introduced through enzymatic modifications post PKS assembly [25]. Such findings yet again highlight the biosynthetic potential of the *Streptomyces* species. It is very likely that the *Streptomyces* sp. (USC-16018), under different laboratory conditions, will be able to produce a wide range of additional secondary metabolites.

**Figure 4:**
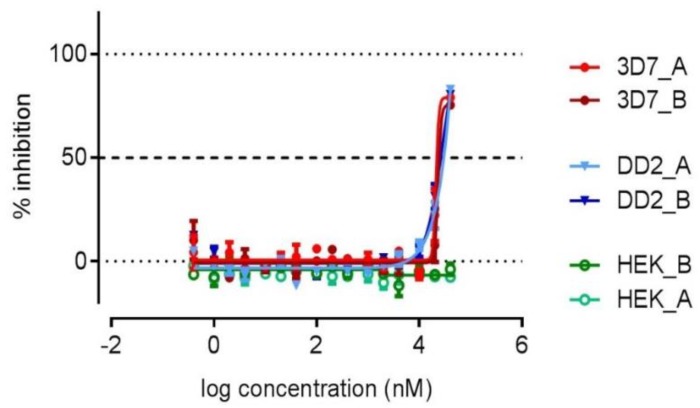
The dose-response curve for **1** against 3D7, Dd2, and HEK-293 in duplicates.

With the aim to discover marine actinomycete-derived antiplasmodial compounds, **1**–**6** were screened for activity against the drug-sensitive 3D7 strain and the chloroquine- and pyrimethamine-resistant Dd2 strain of *P. falciparum* (Table 2). Elaiophylin (**2**) inhibited both the 3D7 and Dd2 parasites with similar potency, IC_50_ = 778 nM and 598 nM, respectively. Antiplasmodial activity in the same range (IC_50_ 214 nM) against the K1 strain was previously reported in the literature [26]. Herbimycin G showed no cytotoxicity and some degree of parasite growth inhibition, corresponding to a 77% inhibition against 3D7 at 40 μM. The inhibition curve did not reach the full inhibition plateau and, therefore, an IC_50_ value could not be determined (Figure 4). The remaining compounds were devoid of antiplasmodial or cytotoxic activity, while the activity of the reference compounds was in the expected range (Table 2).

These results showed that marine actinomycetes were a valuable source for the discovery of new secondary metabolites. Several herbimycin, geldanamycin, and other ansamycin compounds have previously been reported from terrestrial and marine *Streptomyces* species. To our knowledge, the new compound, herbimycin G (**1**), is the first example of such a compound containing a 2,6-disubstituted 4-hydroxyl-cyclohexenone ring. Herbimycin G exhibited weak antiplasmodial activity with no cytotoxicity. We investigated the physiochemical properties of the reported herbimycins and related analogues and found that **1** had a lower cLog*P* compared to the other herbimycin analogues, potentially related to the different ring structure, suggesting a good bioavailability. As herbimycin G did not show cytotoxicity against HEK293, it appears promising to test this compound for other biological activity, such as antitumour activity, in the future.

## 3. Materials and Methods

### 3.1. General Experimental Section

The NMR spectra were recorded at 25 °C on a Bruker Avance III HDX 800 MHz spectrometer with a triple (TCI) resonance 5 mm cryoprobe (Bruker, Billerica, MA, USA). The ^1^H and ^13^C chemical shifts were referenced to the DMSO-*d*_6_ solvent peak at *δ*_H_ 2.50 and *δ*_C_ 39.5, respectively. Standard parameters were used for 2D NMR spectra, including gCOSY, gHSQC, gHMBC, and ROESY. The HR-ESI-QTOF-MS data were recorded on a 6530 Accurate Mass Q-TOF (Agilent Technologies, Santa Clara, CA, USA) workstation using positive electrospray ionisation, with the mobile phase ACN/H_2_O containing 0.1% formic acid. A LaChrom Merck Hitachi L7100 series pump (Tokyo, Japan) equipped with a Hitachi L7450 PDA detector (Tokyo, Japan) was used for HPLC separations. Prior to HPLC separation, the microbial crude extract was adsorbed onto Alltech Davisil 35–75 μm 150 Å C_18_ (Columbia, MD, USA). All separations were performed with a Thermo Hypersil-Keystone Betasil 5μm Å C_18_ HPLC column (21.2 mm × 150 mm) (Waltham, MA, USA). The optical rotations were measured on a JASCO P-1020 polarimeter (Easton, MD, USA). The UV spectra were obtained on a Shimadzu UV-1800 UV/vis spectrophotometer (Kyoto, Japan). The CD spectra were recorded on a JASCO J-715 spectropolarimeter (Easton, MD, USA). All solvents were Scharlau HPLC grade (Barcelona, Spain), except the EtOAc used for extractions was of the Honeywell HPLC grade (Morris Plains, NJ, USA), and for MS, Fisher Scientific LC/MS grade acetonitrile (Hampton, NH, USA) was used. The H_2_O was Millipore Milli-Q PF filtered (Sartorius, Göttingen, Germany).

### 3.2. Biological Material

*Streptomyces* sp. (USC-16018) was isolated from the ascidian *Symplegma rubra* collected on July 15, 2015, Hastings Point, NSW, Australia [9]. The ascidian specimen was identified by Merrick Ekins (Queensland Museum, Brisbane, Australia). The isolate was cultured using selective media and identified via 16S rRNA sequencing. The 16S sequence was deposited at GenBank (accession number MF773774).

### 3.3. Fermentation, Extraction, and Isolation

*Streptomyces* sp. (USC-16018) was grown on 55 OMA (20 g oatmeal, 20 g bacteriological agar, 3 g yeast extract, 1 L deionised H_2_O) and 55 GST (5 g glucose, 2 g starch, 3 g beef extract, 5 g yeast extract, 5 g tryptone, 4 g CaCO_3_, 4 g NaCl, 1 g NaSO_4_, 0.5 g KCl, 2 g MgCl, 0.5 g KH_2_PO_4_, 20 g bacteriological agar, 1 L deionised H_2_O) agar plates for 2 weeks at 28 °C. The plates were then exhaustively extracted by shaking in ethyl acetate. The extracts were dried under a reduced vacuum to yield 1.6 g of dark brown gum for OMA cultures and 0.8 g of light red gum for GST cultures. Each of the extracts was dissolved in a small amount of MeOH and C_18_ silica gel was added in a 1:2 ratio. The solvent was evaporated and the extract adsorbed on the C_18_ was transferred to a refillable HPLC guard column (10 mm × 20 mm) which was attached to a C_18_ Betasil HPLC column. At a flow rate of 9 mL/min, the isocratic HPLC conditions of the 10% aqueous MeOH were initially employed for 10 min, followed by a linear gradient over 40 min from 10% to 100% MeOH, finishing with another 10 min isocratic conditions at 100% MeOH. Sixty one-minute-fractions were collected and analysed by ^1^H NMR spectroscopy. Of the OMA extract, fractions 31–35 were combined and further purified by eluting with a gradient from 25% to 60% aqueous MeOH over 40 min to yield 0.9 mg of compound **1**. Fraction 46 and 26 of the GST extract HPLC separation yielded 0.6 mg and 0.7 mg of compound **2** and **3**, respectively. Fractions 20–25 were combined and repurified to yield 1.2 mg of compound **4** and 2.1 mg of compound **5**. A total of 0.9 mg of compound **2** were also detected in the OMA extract.

Herbimycin G (**1**): C_30_H_46_N_2_O_9_; brown solid; [α]_D_ + 8.6 (*c* 0.007 g/100 mL, MeOH), UV (MeOH) λ_max_ (log *ε*) 245 (1.93) nm; ^1^H NMR (800 MHz, DMSO) and ^13^C NMR (200 MHz, DMSO) data, Table 1; (+)-HR-ESIMS *m*/*z* 601.3111 [M + Na]^+^ (calculated for C_30_H_46_N_2_NaO_9_, 601.3096); all 1D and 2D NMR spectra in Supplementary Material, Figures S3–S8.

Elaiophylin (**2**): C_54_H_88_O_18_; brown solid; ^1^H NMR (800 MHz, DMSO) and ^13^C NMR (200 MHz, DMSO) data, Supplementary Material, Table S2; (+)-LR-ESIMS *m*/*z* 1024.6 [M + H]^+^ (calculated for C_54_H_88_O_18_, 1024.60).

Cyclo-l-Pro-l-Leu (**3**): C_11_H_18_N_2_O_2_; yellow solid; UV (MeOH) λ_max_ (log *ε*) 277 (0.71) nm, 329 (−0.01) nm; ^1^H NMR (800 MHz, DMSO) and ^13^C NMR (200 MHz, DMSO) data, Supplementary Material, Table S3.

Cyclo-l-Pro-l-Phe (**4**): C_14_H_16_N_2_O_2_; yellow solid; UV (MeOH λ_max_ (log *ε*) 216 (1.58) nm; ^1^H NMR (800 MHz, DMSO) and ^13^C NMR (200 MHz, DMSO) data, Supplementary Material, Table S3.

Cyclo-l-Pro-l-Val (**5**): C_10_H_14_N_2_O_2_; yellow solid; UV (MeOH λ_max_ (log *ε*) 215 (1.72) nm; ^1^H NMR (800 MHz, DMSO) and ^13^C NMR (200 MHz, DMSO) data, Supplementary Material, Table S3.

Cyclo-l-Pro-l-Tyr (**6**): C_14_H_16_N_2_O_3_; yellow solid; UV (MeOH λ_max_ (log *ε*) 215 (1.72) nm; ^1^H NMR (800 MHz, DMSO) and ^13^C NMR (200 MHz, DMSO) data, Supplementary Material, Table S3.

### 3.4. Molecular Modelling Calculations

The generation of conformers was performed using the Schrödinger MacroModel 2016 (Version 10.8, Schrödinger, LLC, New York, NY, USA) using the protocol reported by Willoughby and co-workers [27]. First-principle calculations based on the density functional theory (DFT) were carried out to optimize the atomic structures at the B3LYP/6-31G(d) level with the Gaussian 16 (Revision A.03, Gaussian, Inc., Wallingford, CT, USA) suite of programs [28]. The electronic transition and rational strength were calculated using the time-dependent DFT (TDDFT) method at the same theoretical level. The solvent effect in the MeOH solution was considered during all calculations using the Polarizable Continuum Model (PCM) [29]. The Boltzmann-weighted UV and ECD were calculated using the freely available software SpecDis (Version 1.7, Würzburg, Germany) [30] and GaussSum (Version 3.0, Cambridge, UK) [31] using a sigma/gamma value of 0.25 eV. The Lipinski parameters were calculated in ChemBio3D Ultra 14.0 (PerkinElmer, Waltham, MA, USA).

### 3.5. Biological Activity Testing

*P. falciparum* parasites (3D7 and Dd2 strains) were grown in RPMI 1640 supplemented with 25 mM HEPES, 5% AB human male serum, 2.5 mg/mL Albumax II, and 0.37 mM hypoxanthine. The parasites were subjected to two rounds of sorbitol synchronization before undergoing compound treatment. The ring stage parasites were exposed to the experimental compounds in 384-wells imaging microplates (PerkinElmer CellCarrier, Waltham, MA, USA), as previously described in Reference [32]. The plates were incubated for 72h at 37 °C, 90% N_2_, 5% CO_2_, 5% O_2_, then the parasites were stained with 2-(4-amidinophenyl)-1*H*–indole-6-carboxamidine (DAPI), and imaged using an Opera QEHS micro-plate confocal imaging system (PerkinElmer, Waltham, MA, USA). The images were analysed as previously described in Reference [32].

Human Embryonic Kidney cells (HEK293) were maintained in the Dulbecco’s Modified Eagle Medium (DMEM) medium supplemented with 10% FBS. The HEK293 cells were exposed to the compounds in TC-treated 384-wells plates (Falcon, Durham, NC, USA). The Plates were incubated for 72 h at 37 °C, 5% CO_2_, then the media was removed from the wells and replaced with an equal volume of 44 µM resazurin. After an additional 5–6 h incubation at standard conditions, the total fluorescence (excitation/emission: 530 nm/595 nm) was measured using the Envision plate reader (PerkinElmer, Waltham, MA, USA).

The raw data were normalized using the in-plate positive and negative controls to obtain the normalized % inhibition data, which was then used to calculate the IC_50_ values through a 4-parameter logistic curve fitting in the GraphPad Prism (Version 6.0, La Jolla, CA, USA). The experiments were carried out in two independent biological replicates, each consisting of two technical replicates.

## Figures and Tables

**Table 1:** The NMR data for herbimycin G (**1**) (800 MHz in DMSO-*d*_6_, *δ* in ppm).

Position	*δ*_C_	*δ*_H_ (Multiplicity, *J* in Hz)	COSY	HMBC	ROESY
NH		8.02 (d, 9.4)	20	1, 20	3, 19a
1	171.5				
2	133.6				
3	127.5	7.13 (dd,12.2, 1.2)	4	1, 3, 5, 22	
4	126.4	6.48 (ddd, 12.2, 10.9, 1.4)	3, 5	2, 6	
5	135.4	5.65 (dd, 10.9, 7.9)	4, 6	4	
6	78.1	4.56 (dd, 7.9, 1.4)	5, 7	5	7
6-OCH_3_	56.5	3.20 (s)			
7	78	5.16 (s)	6	8	6, 24
7-OCONH_2_	156.5	6.66, 6.30			
8	133				
9	129.4	5.20 (d, 7.7)	10	23	
10	33.5	2.45 (m)	9, 11, 24	8, 11	
11	83.8	3.15 (m)	10, 12	12-OCH_3_, 13	
11-OCH_3_	59.8	3.35 (s)		11	12, 24
12	82.3	3.33 (m)	11, 13		11-OCH_3_, 13a
12-OCH_3_	56.5	3.19 (s)		12	
13a	33.8	1.41 (dddd, 14.2, 9.7, 3.4, 3.4)			12, 14, 15
13b		1.55 (m)			
14	36.6	1.51 (m)	13, 15, 25		13a, 15
15	77.6	4.15 (d, 1.4)	14	13, 15-OCH_3_, 16, 17, 25	13a, 14
15-OCH_3_	57.2	3.13 (s)		15	
16	134.0				
17	149.9	6.65 (ddd, 3.3, 1.9, 1.4)		15, 16, 19, 21	
18	65.1	4.68 (dddd, 10.6, 5.1, 4.7, 2.5)	17, 19		19b
18-OH		5.56 (d, 5.1)		17, 18, 19	19a
19a	39.2	1.96 (dddd, 14.1, 11.8, 10.6, 2.5)	18	18, 20	NH, 18-OH
19b		2.26 (dddd, 11.8, 4.9, 4.7 1.9)	18	17, 18, 20, 21	18, 20
20	53.2	4.55 (ddd, 14.1, 9.4, 4.9)	NH,19	19, 21	19b
21	197.1				
22	12.9	1.84 (d, 1.2)		1, 2, 3	
23	14.2	1.62 (s)		7, 8, 9	
24	17.2	0.94 (d, 6.7)	10	9, 10, 11	11-OCH_3_
25	13.9	0.69 (d, 6.7)	14	13, 14, 15	

**Table 2:** The antiplasmodial activity and cytotoxicity of compounds **1**–**6** and the six reference compounds.

Compound	% Inhibition at 40 µM (IC_50_ in nM)		
	*P. falciparum* 3D7	*P. falciparum* Dd2	HEK-293 Cells
Herbimycin G (**1**)	77.2	81.7	no effect
Elaiophylin (**2**)	96.6 (777.9)	86.1 (598.5)	101.9 (1445)
Cyclo-l-Pro-l-Leu (**3**)	45.9	39.0	no effect
Cyclo-l-Pro-l-Phe (**4**)	no effect	no effect	no effect
Cyclo-l-Pro-l-Val (**5**)	no effect	no effect	no effect
Cyclo-l-Pro-l-Tyr (**6**)	no effect	no effect	no effect
Artesunate	99.6 (0.9)	98.4 (1.3)	50.6
Chloroquine	98.8 (10.0)	96.5 (87.9)	40.9
Dihydroartemisinin	99.9 (0.4)	98.0 (0.6)	37.9
Puromycin	99.0 (148.9)	99.1 (114.4)	102.6 (1409.5)
Pyrimethamine	98.7 (4.7)	23.2	68.4
Pyronaridine	99.9 (7.4)	97.8 (8.3)	98.7 (2825.5)

## Supplementary Materials

The following are available online at http://www.mdpi.com/1660-3397/16/6/189/s1, Figure S1: HSQC-TOCSY NMR spectrum; Figure S2: Herbimycin analogues and related ansamycins; Figure S3–S8: 1D and 2D NMR spectra of **1**; Table S1: Physiochemical parameters of herbimycins and related ansamycin compounds; Table S2–S3: NMR data for **2**–**5**.
