# Supplementary material for: HSQC-TOCSY Fingerprinting-Directed Discovery of Antiplasmodial Polyketides from the Marine Ascidian-Derived Streptomyces sp. (USC-16018)
Source: Mar Drugs. 2018 May 30;16(6):189. doi: 10.3390/md16060189 (PMC6025042; doi:10.3390/md16060189)
Supplement: Supplementary file 1 [file marinedrugs-16-00189-s001.docx]

*Article*

**HSQC-TOCSY Fingerprinting Directed Discovery of Anti-plasmodial Polyketide Compounds from the Marine Ascidian-Derived *Streptomyces* sp. (USC-16018)**

**Larissa Buedenbender ^1^, Luke P. Robertson ^1^, Leonardo Lucantoni ^2^, Vicky M. Avery ^2^, D. İpek Kurtböke ^3;^ and Anthony R. Carroll^1,2,^***

^1^ Environmental Futures Research Institute, School of Environment and Science, Griffith University, Gold Coast Campus, QLD, 4222, Australia; E-mails: larissa.buedenbender@griffithuni.edu.au ; luke.robertson2@griffithuni.edu.au ; a.carroll@griffith.edu.au

^2^ Griffith Institute for Drug Discovery, Griffith University, Brisbane, QLD 4111, Australia; E-mails: l.lucantoni@griffith.edu.au, v.avery@griffith.edu.au

^3^ GeneCology Research Centre, Faculty of Science, Health, Education and Engineering, University of the Sunshine Coast, Maroochydore, Qld 4558, Australia; E-mail: ikurtbok@usc.edu.au

***** Correspondence: a.carroll@griffith.edu.au; Tel.: +61 7 55529187; ikurtbok@usc.edu.au, Tel: +61 7 5430 2881

**Figure S1.** HSQC-TOCSY spectrum of USC-16018 EtOAc extract (DMSO-*d_6;_* 800 MHz).

Figure S2. Herbimycin analogues and related ansamycins.

Figure S2 (*cont.*). Herbimycin analogues and related ansamycins.

| **Compound** | **MW** | **clogP** | **HBA** | **HBD** | **RB** | **TPSA** |
| --- | --- | --- | --- | --- | --- | --- |
| HerbA | 574.289 | 2.1592 | 8 | 2 | 2 | 152.48 |
| HerbB | 530.2628 | 2.156 | 7 | 3 | 4 | 154.25 |
| HerbC | 560.2734 | 1.396 | 8 | 3 | 5 | 163.48 |
| HerbD | 647.2877 | 2.505 | 8 | 4 | 6 | 165.67 |
| HerbE | 590.2839 | 2.107 | 9 | 3 | 6 | 172.71 |
| HerbF | 544.2785 | 3.297 | 7 | 3 | 5 | 138.57 |
| HerbG | 578.3203 | 0.6762 | 8 | 3 | 6 | 155.64 |
| HerbA-2 | 666.264 | 1.977 | 8 | 2 | 8 | 152.48 |
| HerbB-2 | 548.2734 | 0.985 | 8 | 4 | 4 | 174.48 |
| HerbB-3 | 546.3577 | 1.011 | 8 | 4 | 4 | 174.48 |
| TAN420A | 548.2734 | 1.178 | 8 | 6 | 4 | 180.8 |
| TAN420B | 546.2577 | 0.634 | 8 | 4 | 4 | 174.48 |
| TAN420E | 576.3047 | 2.703 | 8 | 4 | 6 | 158.8 |
| HeronA | 651.319 | 1.544 | 8 | 4 | 6 | 167.67 |
| Geld | 560.2734 | 2.0573 | 8 | 3 | 5 | 163.48 |
| 17-AAG | 585.305 | 3.019 | 8 | 4 | 7 | 166.28 |
| Reblast | 546.2941 | 3.305 | 7 | 4 | 5 | 149.57 |
| MacI | 514.2679 | 2.962 | 6 | 3 | 3 | 145.02 |

Table S1. Physiochemical parameters of herbimycins and related ansamysin compounds.

**Figure S3.** ^1^H NMR spectrum of herbimycin G (**1**) (DMSO-*d_6;_* 800MHz).

**Figure S4.** ^13^C NMR spectrum of herbimycin G (**1**) (DMSO-*d_6;_* 800MHz).

**Figure S5.** COSY NMR spectrum of herbimycin G (**1**) (DMSO-*d_6;_* 500MHz).

**Figure S6.** HSQC NMR spectrum of herbimycin G (**1**) (DMSO-*d_6;_* 800MHz).

**Figure S7.** HMBC NMR spectrum of herbimycin G (**1**) (DMSO-*d_6;_* 800MHz).

**Figure S8.** TOCSY NMR spectrum of herbimycin G (**1**) (DMSO-*d_6;_* 800MHz).

**Figure S9.** ROESY NMR spectrum of herbimycin G (**1**) (DMSO-*d_6;_* 800MHz)

Table S2. NMR data for 2 (800 MHz in DMSO-d_6_, δ in ppm).

| **position** | **δ_H_** | **δ_C_** |
| --- | --- | --- |
| 1,1'' |  | 167.1 |
| 2,2'' | 5.70 (d, 15.3) | 121.0 |
| 3,3'' | 6.82 (dd, 15.3, 11.3) | 144.7 |
| 4,4'' | 6.12 (dd, 14.9, 11.3) | 130.4 |
| 5,5'' | 5.65 (dd, 14.9, 10.3) | 144.8 |
| 6,6'' | 2.48 (m) | 40.7 |
| 7,7'' | 5.08 (d, 10.3) | 75.4 |
| 8,8'' | 1.78 (m) | 36.0 |
| 9,9'' | 3.77 (m) | 69.2 |
| 10,10'' | 1.57 (m) | 42.6 |
| 11,11'' |  | 99.2 |
| 12,12'' | 0.96, 2.25 * | 36.4 |
| 13,13'' | 3.78 (m) | 68.8 |
| 14,14'' | 1.05 (m) | 48.1 |
| 15,15'' | 3.76 (m) | 65.6 |
| 16,16'' | 1.04 (d, 6.3) | 18.9 |
| 17,17'' | 0.96 (d, 6.3) | 15.3 |
| 18,18'' | 0.78 (d, 6.8) | 9.0 |
| 19,19'' | 0.86 (d, 6.9) | 6.8 |
| 20,20'' | 1.36, 1.60 (m) | 18.7 |
| 21,21'' | 0.78 (t, 7.4) | 9.0 |
| 1',1''' | 4.91 (d, 3.3) | 91.9 |
| 2',2''' | 1.38* | 32.9 |
| 3',3''' | 3.37* | 65.0 |
| 4',4''' | 3.38* | 70.0 |
| 5',5''' | 3.75 (m) | 66.0 |
| 6',6''' | 1.08 (d, 6.3) | 17.0 |

* Signal overlap and multiplicity could not be clearly defined

| **position** | **3** |  | **4** |  | **5** |  | **6** | |  |
| --- | --- | --- | --- | --- | --- | --- | --- | --- | --- |
|  | δ_H_ | δ_C_ | δ_H_ | δ_C_ | δ_H_ | δ_C_ | δ_H_ | δ_C_ |  |
| 1 |  | 168.0 |  | 165.0 |  | 165.9 |  | 165.0 |  |
| 3 | 3.38, 3.38 (m) | 44.5 | 3.27, 3.38 (m) | 44.6 | 3.26, 3.35 (m) | 44.4 | 3.36, 3.40 (m) | 44.2 |  |
| 4 | 1.83, 1.83 (m) | 22.0 | 1.73 (m) | 21.0 | 1.79, 1.87 (m) | 22.0 | 1.72, 1.84 (m) | 21.7 |  |
| 5 | 1.90, 2.13 (m) | 27.0 | 1.42, 2.01 (m) | 27.8 | 1.84, 2.13 (m) | 27.6 | 1.40, 2.00 (m) | 27.6 |  |
| 6 | 4.19 (ddd, 1.7, 7.9, 9.2) | 59.9 | 4.06 (ddd, 1.2, 6.7, 9.8) | 58.5 | 4.11* | 58.0 | 4.05 (dd, 6.4, 9.0) | 58.9 |  |
| 7 |  | 171.0 |  | 169.0 |  | 171.1 |  | 169.0 |  |
| 8 | 8.01 (s) |  | 7.99 (s) |  | 7.97 (s) |  | 7.86 (s) |  |  |
| 9 | 4.00 (dd, 5.6, 7.6) | 52.3 | 4.35 (ddd, 1.0, 4.9, 5.1) | 56.0 | 3.92* | 59.5 | 4.26 (dd, 4.8) | 55.7 |  |
| 10 | 1.37, 1.76 (m) | 37.0 | 3.02, 3.06* | 35.0 | 2.35 (m) | 27.3 | 2.93, 2.93 (t, 4.5) | 35.6 |  |
| 11 | 1.87 (m) | 23.3 |  | 137.0 | 0.85 (d, 7.1) | 16.4 |  | 127.0 |  |
| 12 | 0.86 (d, 4.5) | 21.2 | 7.27* | 129.0 | 1.01 (d, 7.1) | 18.2 | 7.05 (d, 8.7) | 131.0 |  |
| 13 | 0.88 (d, 4.5) | 22.0 | 7.26* | 127.0 |  |  | 6.63 (d, 8.7) | 115.0 |  |
| 14 |  |  | 7.19* |  |  |  |  | 156.0 |  |
| 15 |  |  | 7.26* | 127.0 |  |  | 6.63 (d, 8.7) | 115.0 |  |
| 16 |  |  | 7.27* | 129.0 |  |  | 7.05 (d, 8.7) | 131.0 |  |

Table S3. NMR data for 3 – 5 (500 MHz in DMSO-d_6_, δ in ppm).

* Signal overlap and multiplicity could not be clearly defined
